# Bridge over Troubled Water: Patients’ Opportunities for Collective Participation in Substance Use Institutions through Research Circles

**DOI:** 10.3390/ijerph182111060

**Published:** 2021-10-21

**Authors:** Brit-Marie Follevåg, Sissel Seim

**Affiliations:** 1 Faculty of Health and Social Sciences, Western Norway University of Applied Sciences, 6851 Sogndal, Norway; brit.marie.follevag@hvl.no; 2 Faculty of Social Sciences, Oslo Metropolitan University, 0130 Oslo, Norway

**Keywords:** collective participation, empowerment, recognition and communication, substance use, research circle, milieu therapy

## Abstract

This study explores patients’ opportunities for collective participation in an institution for people with substance use disorder. Patients and staff from the treatment institution cooperated with researchers to make changes in the treatment practice, using a research circle as a model for the project. In the article, we discuss the following research questions: How and in what areas did patients have the opportunity to participate collectively in the treatment institution? How did the patients experience participation in the research circle? Data consist of minutes from meetings, seminars, and focus-group interviews. The participants analysed the material together, and the authors carried out a thematic analysis after the project. The participants chose to explore how milieu therapy could build a bridge from treatment in the institution to life after treatment, a “Bridge over troubled waters”, to quote Simon and Garfunkel. Findings show that activities in the research circle led to changes at the institution, e.g., regular Sunday afternoon meetings, a weekly quiz, and less controlling procedures of substance use, and that the institutional culture in general became based more on participation and equality. Patients, staff, and researchers participated in a partnership; mutual recognition promoted cooperation and fellowship in the research circle. We conclude that the project provided the participants with opportunities for collective participation in the institution. In addition, the patients experienced partnership and empowerment in the research circle. Our attempts to change institutional practices yielded some improvements but also met with structural and cultural barriers. Thus, the project experienced challenges and obstacles mostly related to limitations in the institutional system and culture.

## 1. Introduction

User involvement is a widely accepted goal in health and social services. Plans and strategies for services for people with substance use problems emphasize that participation is a movement towards openness in public services, increase of legitimacy, and a prerequisite for developing good services [1]. The Norwegian Patient and User Rights Act [2] states that patients in treatment institutions are participants with the right to influence treatment and service development [2]. However, research and public documents point to several challenges in services for people with substance use problems [3,4].

Firstly, research and public documents show a lack of individual participation in institutions for people with substance use problems [3,4,5,6,7]. Substance users in treatment have pointed out that they are not listened to and that power differences and hierarchy prevent participation [8,9]. Research shows that treatment does not prepare patients for transition to life outside the institution. After treatment, patients lack the skills to achieve a meaningful life, including employment, housing, and a social network [3,10]. Skudal et al. highlighted that treatment provided by substance use institutions rarely gives patients the skills they need to master life outside the institution [3]. Their study shows that 72 percent of patients experience little preparation for life after substance use treatment and that 86 percent experience poor follow-up after treatment. In another study, Brorson et al. [11] pointed out that institutions must emphasise patients’ experiences to learn what patients require to cope with life after treatment.

Secondly, even if user participation and user involvement are concepts with positive connotations, the content of the concept is still blurry. Nevertheless, several studies in drug treatment services have found that participatory practices positively impact treatment outcomes and relations between patients and service providers [3,5,6,7]. Rance and Treloar found that participatory practices created opportunities for better communication and that relationships built on participation enhanced the therapeutic alliance and functioned as resources for empowering service users [7]. Karlsson and Borg found that empowerment and regaining control of one’s own life were crucial elements for patients with substance use disorder [12]. Borge and Hummelvoll concluded that milieu therapy, which they describe as “a socio-cultural learning model for understanding patients’ learning processes through therapy and participation in an institutional milieu” [13] (p. 40), can provide opportunities for reflection, understanding, and learning of skills. They emphasise that learning processes must occur in interaction and collaboration with fellow patients and staff [13]. Other practice studies show that language, culture, and discursive processes are essential in determining how patients are understood [14]. It takes time for substance users to change their perceptions from being addicted to substances to becoming participants in society [15].

Thirdly, in services for people with substance use problems, scant attention is paid to collective user involvement to develop and improve services. However, in other fields, research highlights that involving experiential knowledge from users opens new perspectives when developing services [16,17,18,19,20]. There is a demand for more knowledge about how institutions practice participation and the advantages and disadvantages of different practices [21], for methods or strategies for collective user participation [22] and dialogue between professionals and users [23].

To meet the need for more knowledge about collective participation in substance use treatment, we developed an action research project in collaboration with patients and staff at a substance use treatment institution. We used a research circle as a strategy for collective participation. A research circle is an arena or a forum for joint knowledge production in a dialogue between the participants in the circle, often with participants from the practice field and academia. The basic idea is to study questions from the participants as a foundation for negotiations in the circle [24].

## 2. Theoretical Perspectives

### 2.1. Collective Participation and Empowerment

In line with current discourses in participatory practice, we understand patients as actors with resources to develop the services they use [8,9,12,25]. User participation means that patients and users of the services are involved in decision-making processes. We use the term *collective* participation when the purpose is to improve the collective good [22,26,27], improving services for everyone in the same situation. In the research circle project, participation has a collective goal of achieving improvement for all patients in the institution.

Arnstein maintains that individual and collective participation requires participants to have power and influence decision-making [28]. Arnstein defines counselling or consultation as tokenism and emphasises that genuine participation must include *a partnership* with opportunities for negotiation and influence or *delegated power or control* [28].

The term empowerment includes awareness of power relations and oppressive mechanisms and strategies for achieving power to improve living conditions. Empowerment of groups refers to raising awareness of a group’s situation and their opportunities for collective action, as in Freire’s understanding of the pedagogy of oppressed people [29]. As Gutiérrez [30] and Rappaport [31] described, empowerment can refer to processes and goals or the result itself. Collective participation and group empowerment may also result in individual empowerment because of enhanced awareness of one’s situation, resources, and values [19,30].

The terms user-participation and empowerment are criticised for being tokenism and rhetoric. There is a risk in collaborations between researchers, professional practitioners and patients that empowerment and participation can reproduce oppressive practices if the collaboration does not involve reciprocity and the opportunity to influence results [6,28,32].

In this article, we use the concepts of collective participation and empowerment to discuss if the patients’ involvement can be understood as genuine participation, as a partnership, or merely as tokenism without power or influence.

### 2.2. Recognition and Institutional Culture

Recognition and respectful communication are central themes in the research circle model. The concept of recognition involves having the ability to place oneself in the other’s shoes, to understand, respect, confirm, and to have emotional availability [33]. The relation is dynamic in the interaction between parties, in this context, between patients and professional practitioners, aiming to bring about the involvement of others. In collective interaction, we are seen and recognised as subjects, contributing to our awareness of ourselves. Recognition is about understanding and valuing the other’s experience to develop mutual understanding and not exercising power over others [33]. The ability to see oneself, see the other, and reflect on actions, provides the strength to draw out something in the other and negotiate [33] (p. 149). When equal parties with different experiences and knowledge talk together, different perspectives challenge and contribute to development.

The culture in a treatment institution has unique characteristics and frameworks. Based on a study of psychiatric hospitals, Løchen [34] (p. 122) described a controlling and restrictive institutional culture with norms and structures that convey no interest in the patients as human beings or room for constructive relations between patients and staff. Løchen [34] refers to two different treatment ideals: Institutions with a controlling and restrictive culture and institutions with a close and interpersonal culture. He points out that treatment institutions usually are organised as hierarchies. First, power is held by doctors and management, and then by personnel in the milieu, patients are at the bottom of the hierarchy. Research on dropout from substance use treatment institutions emphasises the importance of institutional culture. McKellar et al. [35] and Brorson et al. [11] found that patients had a lower risk of dropout from environments they perceived as supportive than from environments they experienced as rigid and controlling. Brorson et al. concluded that therapists should involve patients in decisions about their treatment and establish a good alliance with them [11] (p. 1020).

The concepts of recognition and institutional culture are central elements in discussing patients’ experiences of the research circle and the opportunity to implement changes in the institution.

In this article, we explore the opportunities and challenges for collective participation, drawing on the experiences from the research circle project. We will discuss the following research questions: How and in what areas did patients have opportunity to participate collectively in the treatment institution? How did the patients experience participation in the research circle?

## 3. Materials and Methods

The participants in the research group agreed on participatory action research (PAR) as a research approach. In PAR, knowledge production combines research activities and action for change [36,37,38]. As mentioned, we chose to use a *research circle* as a model for trying out collective participation in cooperation between patients and staff from a treatment institution for people with substance use problems and researchers from the Western Norway University college (HVL).

### 3.1. The Research Circle Project

A research circle is an arena in which practitioners, service users and researchers meet to immerse themselves in a problem of common interest and contribute to change [24]. The research circle project lasted for 1.5 years, from 2013 to 2015, allowing for dialogue and continuity. A total of 16 people participated in the research circle. Six institution staff, four male nurses, one female pedagog and one female psychologist participated throughout the study period. Ten patients at the treatment institution participated in the project. Due to the absence of leave, short stays at the institution, and death, the number of patients varied. The patients who participated were men aged 24–50 who had 5–37 years of experience as substance use patients. Four female researchers from HVL participated, one nurse and three social workers, including the authors.

The participants in the research circle met on three occasions at two-day seminars at a hotel, including an overnight stay. The start-up seminar was followed by an exploration *phase* where we explored the problem areas and topics that the project could examine. After the second seminar, in the action phase, we experimented with changes at the institution. Each phase consisted of six half-day meetings at the institution.

In the start-up seminar, staff, patients, and researchers were actively involved in the process of arriving at topics for the project. The group chose topics from the patients’ narratives of their daily lives outside the institution and from the patients’ and staff’s experiences inside the institution. In addition, the researchers introduced relevant theoretical perspectives into the discussion perspectives of collective participation, empowerment, and recognition. The selected theme for the exploration phase was: How can we strengthen milieu therapy through user participation? Staying together at the hotel, away from day-to-day life in the institution, had created an informal atmosphere, and we got to know each other in new ways. The atmosphere and commitment of the start-up seminar continued in the following seminars and meetings.

In the mid-term seminar, members agreed on themes related to the quality of the milieu in the institution and to develop necessary skills for life after treatment. We agreed on the following vision for the action phase: to create a treatment model to improve the milieu-therapy inside the institution and learn skills that would strengthen the patients’ ability to master life outside the institution after treatment. Referencing Simon and Garfunkel, the patients called this “Bridge over troubled waters” [39].

In the action phase, we attempted to implement changes at the institution. To anchor the work in the treatment institution and HVL, we made information about the proceedings in the research circle available on the participants’ networks, and we discussed proposals for changes to patients and staff in the rehabilitation department.

In the final seminar, we analysed the work of the research circle and discussed how the institution could continue with patient participation.

### 3.2. Data Collection

The data consist of comprehensive and detailed minutes from three seminars and 12 meetings in the exploration and action phases. In addition, the researchers at the final seminar conducted two focus group interviews with patients and staff (separately). Questions posed in the focus groups were related to evaluating the process and content of the research circle and the attempts to make changes in the institution. Questions were also related to patients’ and staff’s motives for participation and their experiences in the research circle. The researchers wrote minutes from the seminars and meetings and transcribed audio recordings from the focus group interviews.

### 3.3. Ethics

We clarified with The Norwegian Centre for Research Data (NSD) that we were not required to register the project with NSD. All participants signed a consent form that includes their approval to participate and accept their mutual duty to maintain personal information confidentiality. We have anonymised all personal information and have not published the institution’s name, even though the institution initially wanted to include the name.

### 3.4. Roles

In PAR, all actors ideally are involved in knowledge production through participating in change processes and dialogue [40]. The participants in the research circle were involved in designing the project and developing the topics and research questions. They were actively involved in the change processes and analysis in the final seminar. The initial idea was that patients and staff also should be co-researchers throughout the analysis and dissemination processes. However, patients and staff did not want to participate in the final analysis or co-author the article. The interpretation and understandings expressed in the paper are, therefore, the authors.

### 3.5. Data Analysis

The participants started developing the research questions inductively at the first seminar. We carried out the first analysis when the participants discussed and approved the minutes from seminars and meetings. This round of analysis constitutes a participant validation of the material [41,42]. The questions in the exploration phase centred around user participation. New questions and themes were added and explored through discussions during the meetings in the research circle. At the final seminar, the group analysed the material from the research circle. We concluded that the central theme in the work of the research circle had been to make changes in the institutional milieu to strengthen the mastery of life outside, in the participants’ words, to create a *Bridge over troubled waters*.

For this article, the authors applied a thematic analysis [43,44] of the two data sets: written minutes from meetings and seminars and transcribed recordings of focus group interviews with patients and staff. We followed the steps in a thematic analysis outlined by Braun and Clarke [43] (pp. 87–93). First, we reread the material, systematically coding exciting topics across the data sets. We then searched for potential themes and connected data relevant to each potential theme. Reviewing the themes, we defined and named themes and subthemes. For producing the report, we identified quotations as examples for the themes and subthemes. The final part of the analysis was also theoretically informed. We analysed the themes using the theoretical frames of collective participation, empowerment, and recognition. We identified that one central theme is opportunities for making changes in the treatment practice through collective participation, and we identified three subthemes: 1. Mastering practical skills, 2. Recognition and respectful communication, 3. Trust and cooperation. Another central theme is the patients’ experiences as participants in the research circle, sub-themes 1. Recognition and 2. Dialogue.

## 4. Results

### 4.1. Opportunities for Making Changes in the Treatment Practice through Collective Participation

#### 4.1.1. Mastering Practical Skills

The patients believed that milieu therapy must include learning skills to master daily life when they leave the institution. “Freedom is threatening”, the patients said. They pointed out that they had to learn many skills to master life outside the institution, one said:

“I am not ready for all the situations I end up in when I come out. I am getting no help here to master the life out there. How could we train for the outside in here: tidy your room, cooking, teach me how to behave in a job interview, enjoy my own company, how to behave when I want to rent somewhere to live when taking a phone call? Instruction in everyday tasks.”

Crucial areas were cooking and tidying their rooms. The patients wanted to take responsibility for the kitchen service at the institution, buy the food, cook it, lay the table, and clean up afterwards. The patients thought that the kitchen should be an area in which patients and staff cooperated. One said: “Cooking, creating a sense of community around the table is important. It is crazy that a table is hardly laid for breakfast and lunch”.

The patients said it was challenging to have responsibility for specific tasks like tidying their rooms and cooking. They wanted to be challenged to take collective responsibility and to receive individual feedback on their performance. They thought it would increase their self-respect and that they would become more aware of their weaknesses and strengths.

#### 4.1.2. Recognition and Respectful Communication

At the up-start seminar in the research circle, it emerged that recognition and respectful communication are essential elements in achieving personal mastery, security, and trust. The patients’ experience from the research circle was that everyone listened to each other irrespective of agreeing or disagreeing. As a result, patients and staff felt it was essential to transfer respectful communication to the environment in the institution.

The patients explained that they lived in a hierarchy outside the institution’s environment but continued living in a hierarchy in the treatment system—again in the lowest caste. They were concerned about the lack of respectful communication and participation. The participants agreed that respectful communication should be a goal but that it should at the same time be direct and confrontational.

The staff experienced the institution’s culture as more of control than trust and saw a need to change the interaction between staff and patients. The discussion about control and trust took as an example the institution’s procedure of requiring patients suspected of using substances to submit a urine sample. The staff thought it was better to be open and confront the patient directly with suspicion. The patients answered that they needed openness and directness, one saying: “We want, and we tolerate direct and challenging communication”. They emphasised that the staff should give honest and constructive feedback: “We must be able to correct each other and still maintain our relationships”.

#### 4.1.3. Trust and Cooperation

The patients believed that community and good relationships between patients and staff were vital in building and maintaining trust and cooperation. They also emphasised that patients are essential to each other in the institutional environment. They understand each other and know what can trigger unease or promote a feeling of security. Therefore, the patients in the research circle established a regular meeting for all patients every Sunday to promote a sense of community in the institutional environment. The purpose of these meetings was to share ideas about activities in the research circle, receive input from other patients, and implement proposals for change in the environment. In addition, the patients set up an ‘activity bank’ in the department to summarise how the week had gone, determine what they could do differently, and make plans for the next week.

The patients felt insecure in social settings; they were not used to talking about anything other than substances and were poorly informed about current affairs. However, one patient said: “Knowing a little about what has been in the news can be useful when trying to find topics to talk about with others”. Therefore, patients and staff agreed to plan and arrange a quiz once a week. The intention was to increase everyday knowledge, promote social engagement, and create a social community among the patients and staff. One said a quiz would broaden their horizons: “It will make us open the windows a little and let the outside in.” To arrange and participate in a quiz would challenge the patients to work together and to focus and remember.

In the project period, patients and staff tried out quizzes, cooking projects, and interviews when there was suspicion of intoxication. Patients and staff agreed that these changes were good examples of participation and active milieu work and could increase patients’ ability to master life outside. These changes were, however, not permanently established as practices at the institution.

### 4.2. The Patients’ Experiences as Participants in the Research Circle

#### 4.2.1. Recognition

The patients said they soon felt that everyone recognised each other in the research circle. Being recognised and perceived as equals strengthened their self-confidence, leading to personal growth and an experience of mastery. One said:

Easy to take part in equal. It was like this right from the start: managers, staff, and patients. Everyone talking, everyone respected. No matter who. A fantastic way to work. When we enter treatment, we do not expect to sit talking to the management and top academics. We have gained great self-respect from this.

The patients thought the different perspectives contributed to the good dialogue. One said: “Many different people sitting together and chatting—a lot of good comes out of it!” The research circle allowed the staff to see that the patients have the resources and strength to contribute and that drawing out the patients’ perspectives is essential. “What the patients bring up here, their experiences, gives me new knowledge, experience-based knowledge that I can process and use in other settings”.

#### 4.2.2. Dialogue

Several patients said they had not previously dared to speak in groups, but participation in the research circle had changed this. The patients emphasised that they could be open and honest with other patients, they spoke the same ‘language’, and that they in the research circle experienced the same form of dialogue with the staff and researchers. They, however, reflected upon that cooperation with the staff at the institution was often based upon insecurity and misunderstandings.

All patients, staff, and researchers commented that the interaction in the research circle helped remove the barriers between participants. For example, one patient described their interaction with the staff members in the research circle as being: “A fantastic way to break down barriers. That does not mean we respect them less. No one will lose their role because they have breached these boundaries”. The staff said that the close interaction in the research circle was a new experience; it had been helpful in the research circle to get close to the patients, hear their experiences and perceptions, and collaborate with them.

The patients thought the conversation form in the research circle could be helpful in treatment. Daring to express oneself and getting good feedback afterwards gives a feeling of mastery. It is, however, not problem-free. As one said: “It can, at the same time, be more difficult because you are naked without intoxication, have more shame, which can make it more difficult to deal with”. All participants, patients, staff, and researchers experienced the research circle as a new experience of individual and collective participation, contributing ideas, and reflections.

## 5. Discussion

### 5.1. Opportunity for Collective Participation in the Treatment Institution

Did the patients have opportunities for collective participation in the treatment institution? What promoted and what hampered participation opportunities?

The patients’ stories represented the starting point for choosing the themes that led to attempts for change in the institution. Their stories told about their experiences in society after previous treatment programs: their difficulties in mastering the transition to life without substances, a lack of practical day-to-day life skills, a lack of social networks and problems communicating with others. The patients had experienced that the milieu therapy provided by the institution did not help strengthen the skills they needed to master everyday life. The patients’ descriptions align with other research, showing that substance use treatment institutions do not plan well for discharge and transition from institutions to life outside [3,6,10].

The patients’ stories were the starting point for discussions on changing milieu therapy in the action phase. Proposal formation was, however, a joint project in which all participants actively participated.

Several change proposals were implemented in the institution to create a more supportive environment. One implemented change was a less controlling procedure when the staff suspected patients of using substances of having a dialogue with the patient instead of demanding a urine sample. The institution staff became aware, during the project, of the challenges of their role as guards, exercising control, and at the same time attempting to create good relationships and build trust.

Other changes were intended to improve the sense of community and social training in the institution. Patients got responsibility for the activity bank and the Sunday evening patient meetings. Patients and staff worked together on activities, arranged the quiz, and became more present in the department. The changes provided patients with communication and interaction training during the execution of these tasks. The changes were intended to achieve closer cooperation in the patient group and between patients and staff. Delegating responsibility for tasks and cooperating in their execution positively influenced the culture of the environment and promoted personal development. Other research also emphasises the potential for learning and personal development in institution environments [11]. Therapeutic communities can create cohesion and cooperation and achieve improvement process goals [13]. Seeing each other, respecting different opinions, and recognising others as significant participants in the system are essential aspects of the change process [33]. Eide and Nesvåg pointed out that the content of the milieu therapy is essential for how patients experience day-to-day life after treatment [15].

We can sum up that the exchange of experiences and dialogue in the research circle resulted in constructive proposals for change. Collective and individual participation requires the system and helpers to share power and create a culture of trust and mutual respect: To treat patients as subjects and have faith in their resources and competence to solve problems.

#### Frameworks That Hinder User Participation

The participants in the research circle experienced challenges when trying to implement lasting changes in the institution. The patients’ wish to learn about cooking, which the staff supported, was limited by organisational issues. It was difficult to reconcile to give patients responsibility for cooking and cleaning with the current management firm. The institution used a centralised food supply, which made patients shopping for and cooking food difficult. Making patients responsible for cleaning was challenging to combine because cleaning staff were employed to do this job. Implementing the research circle’s improvement suggestions proved challenging to achieve in practice. The patients felt that that participatory practice had a long way to go and that they still were at the bottom of the treatment institution hierarchy. Their feelings agree with Løchen’s description of a controlling and restrictive institutional culture [34].

We conclude that the patients’ involvement in collective participation, only to a small extent, contributed to changes in the institution’s treatment practices. Viewed as collective participation in the institution’s context, using Arnstein’s conceptual framework [28], we do not understand the patient’s participation as a partnership but as tokenism without power or influence. The challenges presented by the attempts at making changes in the institution are related to the institution’s culture, structure, and forms of governance [35], and the inherent power relations [28] in the institution. In afterthought, we can ask if these challenges may also relate to that we had not sufficiently anchored the research project in the institution.

### 5.2. Experienced Participation and Empowerment in the Research Circle

The good relations between the participants in the research circle, based on recognition and equality, and the close and respectful communication that grew out of this, contributed to an open and constructive discussion of complicated issues. There was also space in the research circle for confrontation, criticism, honest feedback, humour, informal chat, and good stories. The relations contributed to the participants feeling that the boundaries between role and position dissolved. Other research emphasises that patients feel more recognised and valued when professionals show a more personal side [7,13,35]. Changed relations are typical experiences where users, staff, and researchers meet with a common agenda of participation [7,19,45]. The place for meetings is also essential. New surroundings outside the usual treatment setting contributed to greater openness and access to participants’ resources and knowledge [8,46,47].

The sense of community and dialogue in the research circle was a distinctive strength that promoted sharing experiences. Mutual recognition and trust meant that communication expanded perspectives and provided new knowledge. The lack of power struggle is consistent with the findings of appreciative communication research [33]. The participants were open to each other’s stories and experiences. They experienced dialogical cooperation and a mutual understanding of the research circle’s experiences, perspectives, and perceptions. Having time and respect to understand the other as they experience themselves became an essential part of the collaboration. The experiences correspond with findings in the research by Hansen et al. [48] that the inner understanding in respectful communication creates the opportunity to develop shared meaning and prevent misunderstandings, providing action competence. The experiences also concur with research into interaction and respectful communication, showing that recognition, cooperation as equals, and participation can provide an impetus for change and development [7,13,33,49,50].

The interaction between patients, staff, and researchers in the research circle contributed to discovering new aspects of themselves and others. The focus was listening to others, changing language, and expressing feelings without hostile reactions from the other participants. The patients felt that they had to become more aware of communicating their views and opinions in the institutional environment. They wanted to transfer their experiences from interaction in the research circle to everyday life in the institutional environment.

We can summarise that the patients experienced participation in the research circle. They experienced being heard and taken seriously. Their stories and experiences formed the starting point of deciding which themes and issues would become central in the exploration phase. In the action phase, all participants influenced the proposals for change in milieu therapy. We understand the interaction between patients, staff, and researchers in the context of the research circle as a *partnership* where the parties involved can negotiate and influence the results [28].

The patients described their experiences in the research circle as empowering, in the way that Freire [29], Gutiérrez [30], and Rappaport [31] have described the processes of empowerment. The patients experienced that their stories about substance use treatment and their needs after treatment led to proposals for changes in the institutional environment. They said that participating in the research circle contributed to increased self-confidence in raising awareness about their situation. The patients’ motives also related to the fact that the research circle should collectively lead to improvements for others: “This is the driving force behind being here. That commitment, a wish to participate goes onwards and beyond ourselves”. They also stated that this gave them hope for the future, that their efforts could help improve their situation and the situation of other patients in treatment.

### 5.3. Limitations

One limitation of the study is that the patients did not participate in the final analysis and preparation of the article. However, after the project, when two patients and three staff, participated in the dissemination of *Further and master’s education* in HVL, we checked that our understanding concurs with their understanding. Another limitation is that the research did not include information on implementing changes in the institution over time. Further research should include this as part of the evaluation of results.

## 6. Conclusions

We can conclude that the activities in the research circle led to some changes at the institution: Some improvements were implemented in the milieu therapy, and the institutional culture became based more on participation and equality. In addition, the patients experienced partnership and empowerment in the research circle.

To implement proposals that could lead to lasting changes in the organisation and work structures was more challenging. The organisation of the environment and treatment in the institution appeared to be fragmented and divided into different roles, different tasks, and interpretations. For milieu therapy to develop to provide the strength, substance users need to master life outside the institution, all participants in the system, patients, and staff, must work together to change attitudes and working methods. The institutional culture must change from power and control to a humane culture of closeness and participatory practice, as mentioned in other studies [7,11,35]. The previous study from the institution, Larsen and Sagvaag, described the institution as being dominated by a diagnostic culture that hindered participation [6]. They found that staff and managers believed that management permitted empowerment if they found this to be expedient, even though they believed patient participation was valuable.

We conclude that there is a need to change attitudes towards patients with substance use problems, to view them as people who possess competence and resources. It is about time for a paradigm shift in policies and practice in the treatment of substance use problems and the education of the relevant professions. We emphasise three essential prerequisites for making fundamental changes in attitudes, policies, and treatment practices. Firstly, that patients are involved both in individual and collective participation. Secondly, to meet in arenas outside the institution, in places that provide space for an open dialogue between patients and staff. Thirdly, developmental and research projects must be well rooted in the institution’s management to ensure the implementation of suggestions for changes in the organisation, work structures and treatment practices. Finally, we recommend conducting research and development projects in collaboration between the practice fields, patients and professional practitioners, and research and professional education in academia.

We end by quoting one of the patients: “If this can be done, be systematised, disseminated, and benefit others, then this is good. After so many years of rising and fall, this is what one hopes for”.

## Data Availability

The data are not publicly available due to ethical restrictions.
